# The two faces of antibiotics: an overview of the effects of antibiotic residues in foodstuffs

**DOI:** 10.1007/s00204-024-03760-z

**Published:** 2024-04-30

**Authors:** Merve Güdül Bacanlı

**Affiliations:** Department of Pharmaceutical Toxicology, Gülhane Faculty of Pharmacy, University of Health Sciences Turkey, 06018 Ankara, Turkey

**Keywords:** Antibiotic, Food animal, Antibiotic resistance, Toxic effects

## Abstract

Antibiotics, which have been used for many years to treat infections, also play an important role in food contamination with antibiotic residues. There is also unnecessary use of antibiotics, particularly to increase production efficiency. Non-compliance with withdrawal periods and maximum residue limits (MRLs) for antibiotics used in food-producing animals results in undesirable events, such as allergic reactions, teratogenicity, carcinogenicity, changes in the microbiota and, in particular, antibiotic resistance. Therefore, it may be useful to avoid unnecessary use of antibiotics, to limit the use of antibiotics and to turn to alternatives that can be used instead of antibiotics. The aim of this review is to provide information on the undesirable effects of antibiotic residues in food-producing organisms and in the environment, their determination, and the precautions that can be taken.

## Introduction

Antibiotics are naturally occurring, semi-synthetic, and synthetic compounds with antimicrobial activity that can be administered orally, parenterally, or topically and are used in human and veterinary medicine to treat and prevent disease and to promote growth in food animals (Phillips et al. 2004). Since their introduction in the 1930s, antibiotics have primarily been used to treat or prevent human and animal diseases. In addition to their therapeutic value, the discovery in 1950 of their ability to improve growth and feed utilization in food animals led to their widespread use as feed supplements (Chen et al. 2019). Besides, the growth-promoting effects of antibiotics were discovered in the 1940s, when animals fed dried *Streptomyces aureofaciens* mycelia containing residues of chlortetracycline were observed to improve their growth (Bacanlı and Başaran 2019). Indeed, antibiotics can increase growth rate by thinning the mucous membrane in the gut, altering intestinal motility, creating favorable conditions for beneficial gut microbes by destroying harmful bacteria, and splitting proteins for muscle growth. They also promote growth by reducing the activity of the immune system and reducing nutrient wastage and toxin formation (Darwish et al. 2013). Generally, animals receiving antibiotics in their diet gain 4–5% more body weight than those not receiving antibiotics (Bacanlı and Başaran 2019).

Antibiotics are one of the most effective drugs for the treatment of diseases, but their overuse will lead to changes in the gut microbiota and antibiotic resistance (Li et al. 2019; Rahman et al. 2021). Both epidemiologic and experimental studies have suggested that the effects of antibiotics can be cumulative in humans and thus one can expect that the effects of environmental antibiotic exposure on the human microbiome can accumulate over generations (Sonnenburg et al. 2016).

Scientists have proven in recent years that exposure to low doses of antibiotics is associated with many human health problems, including obesity, carcinogenicity, reproductive effects, and teratogenicity. However, some independent reviews tend to believe that "the actual risk is extremely small and could be zero" because very little evidence directly shows the health hazards caused by exposure to low doses of antibiotics in food (Chen et al. 2019). In addition, overuse of antibiotics can lead to the development of resistance, which can directly and indirectly affect bacterial populations and result in failure to treat infections. In fact, it is mainly emphasized that antibiotic residues in food cause these toxic effects (Shahid et al. 2021). Although the effects of antibiotic residues on human health are predicted, the potential harm to environmental health is generally unknown (Ngangom et al. 2019).

It is aimed to provide information on the use of antibiotics, the toxic effects of antibiotic residues in food and precautions within the scope of this review because antibiotics have such harmful effects as well as beneficial effects, that is, they have two different faces.

## Data collection

Information on antibiotic residues and toxic effects was obtained through literature searches in databases, such as Google Scholar, Pubmed, Sciencedirect and Scopus. “antibiotic”, “antibiotics in food”, “antibiotic residue”, “antibiotic residues in food”, “antimicrobial drugs in food”, “antimicrobial drug residues in foods”, “drug residues in food” were used as keywords.

While conducting the literature search, care was taken to select articles published in the last 10 years in this literature review. In particular, articles on antibiotic residues in foods of animal origin were used. These studies were evaluated to include those examining the undesirable effects caused by antibiotic residues. However, literatures examining the analysis of antibiotic residues in detail were excluded.

## Antibiotics used in foodstuffs

### Antibiotic groups

Antibiotics are often complex molecules that can have different functions within the same molecule. Therefore, under different pH conditions, antibiotics can be neutral, cationic, or anionic. Antibiotics can be grouped according to their chemical structure or mechanism of action. They are divided into different subgroups, such as beta-lactams, amphenicols, tetracyclines, macrolides, aminoglycosides, fluoroquinolones, and others (Ngangom et al. 2019).

Approximately 80% of animals used in food production are treated with veterinary medicines during part or all of their lives (Pavlov et al. 2008). Narrow-spectrum antibiotics should be the first choice of antimicrobial agents (Shahid et al. 2021). Antibiotics commonly used for prophylaxis, growth promotion, and treatment in food animals are sulfamethoxazole, enrofloxacin, benzylpenicillin, tylosin, amoxicillin, trimethoprim, oxytetracycline, ampicillin, and streptopenicillin; amikacin, neomycin, enrofloxacin, doxycycline, tetracycline, tilmicosin, and colistin sulfate (Chattopadhyay 2014; Redwan Haque et al. 2023).

### Reasons for antibiotic use in foodstuffs

The basic aim of antibiotics is to kill or destroy cells that rapidly invade the body as a result of infection, such as mastitis, arthritis, respiratory diseases, gastrointestinal, and other bacterial infections (Darwish et al. 2013). These antibiotics can be given to prevent disease or during an infection.

For an antibiotic to be used in animals, it must have certain properties. It must be safe and effective when used on a large scale. The drug should not be used in human or veterinary medicine, although exceptions are justified to solve serious problems, cause the emergence of resistant strains or cause cross-resistance to other drugs. It should not interfere with other drugs used as a substitute for good hygiene and should not have undue effects on the body's flora (Landers et al. 2012). When antibiotic treatment is necessary, it is usually administered to animals in feed or drinking water (Phillips et al. 2004).

According to various researchers, reliable data on amount and pattern of use of antibiotics, and dose and frequency are not very accurate (Diana et al. 2019). Preventing disease is more effective than treating it. Drug treatment, especially antimicrobials, should never be used as a substitute for good husbandry, biosecurity, and management practices. Excessive weight loss at the beginning of lactation increases susceptibility to disease by causing stress, so medicated feeds are used for therapeutic and preventive purposes in animals. The US Department of Agriculture reported that about 88% of growing pigs receive antibiotics, usually tetracyclines or tylosin, in their feed to prevent disease and promote growth (Ghimpețeanu et al. 2022).

The use of antibiotics throughout an animal's life can significantly increase weight gain by increasing nutrient utilization in the diet, ultimately reducing feed costs, and shortening feeding times (Erofeeva et al. 2021). Antibiotics used as growth promoters do this through mechanisms, such as reducing the incidence and severity of bacterial infections, delaying the uptake of nutrients by microorganisms, reducing the secretion of growth inhibitory metabolites by Gram-positive bacteria, and improving nutrient absorption by thinning the intestinal wall (Ishikawa et al. 2018).

European Union (EU) Regulation No 6/2019 lays down the rules for the authorization of the use of veterinary medicinal products in feed, including the production, distribution, advertising, and supervision of such products. Feed business operators involved in the manufacture, storage, transport, or placing on the market of medicated feed and intermediate products must be authorized by the competent authority in accordance with the authorization system to ensure both feed safety and feed traceability. The labelling of medicated feed should comply with the general principles laid down in EU Regulation No 767/2009 in order to provide users with the information necessary for the correct administration of medicated feed and should be subject to specific labelling requirements. The relevant legislation provides for the establishment of additional instructions for the cleaning of equipment used for the administration of the medicinal products concerned to prevent cross-contamination and reduce antimicrobial resistance (Ghimpețeanu et al. 2022).

### Antibiotic contamination and residues in foodstuffs

There are essentially two main sources of antibiotic residues in food: the subjective use of antibiotics by humans (prevention and treatment of disease) and the non-subjective accumulation of antibiotics in food-producing animals (prevention and treatment of disease as well as growth promotion and improvement of feed efficiency) (Chen et al. 2019). Antibiotics can have serious effects on human health and this has led to the introduction of maximum residue limits (MRLs) in food safety legislation (Ghimpețeanu et al. 2022).

Antimicrobials can accumulate as residues in body tissues, and it takes time for the residues to be excreted or metabolized. The amount of these residues can be particularly high when these animals are consumed by humans during drug treatment or immediately after drug discontinuation (Tollefson and Miller 2000).

Some antibiotics prescribed to poultry and livestock are generally not absorbed from the gut and are mostly excreted without conversion to metabolites (Rafiq et al. 2022). Various sources contribute to this pool of antibiotic residues, such as livestock, dairies, veterinarians, pets, poultry, hospital waste disposal, animal feces, pharmaceutical factories, and municipal waste (Obayiuwana et al. 2018). These residues and metabolites indirectly contaminate water and soil; for example, a polyether antibiotic, monensin, used in dairy farms to stimulate animal growth, has been found to spread to natural sources (Shahid et al. 2021). Therefore, these compounds can enter the human food chain from the environment (Rafiq et al. 2022).

At least three routes are responsible for antibiotic residues in livestock and poultry products, including meat and poultry products: (i) direct injection [e.g. intramuscular (i.m.), intravenous (i.v.) or subcutaneous (s.c.)] for disease prevention and treatment, (2) direct uptake from feed and water where antibiotics are used as additives to promote growth and improve feed efficiency, and (3) topical contact or intramammary or intrauterine infusion (Mitchell et al. 1998). In relation to seafood, antibiotics may also be used as therapeutic and prophylactic agents to treat and prevent disease and as feed additives to promote growth and improve feed efficiency, which may be an important contributor to the availability of antibiotics in commercial fish and shellfish products (Liu et al. 2018).

Various antibiotics in the environment are taken up by plants and some antibiotics even accumulate in plants. Although the consumption of antibiotics in plants is very low compared to animals, residues have been found in water used in agricultural areas, leading to contamination of agricultural land (Piña et al. 2020). The most common vegetables that accumulate antibiotics are cereals, such as wheat, rice and oats, and coarse grains, such as maize and barley, which are thought to transmit antibiotic residues to other living organisms through the food chain (Ghimpețeanu et al. 2022).

Antibiotics used in livestock can also be transferred to fields through fertilizers, and these antibiotic residues can be absorbed into the soil and reach groundwater (Ishikawa et al. 2018). Besides, antibiotics are excreted by humans and animals into wastewater, which can then be carried into rivers and oceans (Shahid et al. 2021).

### Toxic effects of antibiotic residues in food stuffs

Although antibiotic residues in food cause allergic reactions, teratogenicity, mutagenicity, and carcinogenicity, the most important undesirable effect is that they cause antibiotic resistance. Table 1 summarizes the antibiotics with the highest levels of residues in food and their adverse health effects.

**Table 1:** Antibiotics with the highest levels of residues in food and their adverse health effects

	Adverse Health Effects (Ngangom et al. 2019)
Oxytetracycline	Blood changes (leukocytosis, atypical lymphocytes, pulmonary Congestion, toxic granulation of granulocytes, and thrombocytopenia purpura) Damage calcium-rich organs, such as teeth and bones, Cause erosion of the nasal cavities
Erythromycin	Vomiting Teratogenicity
Enrofloxacin	Chromosomal damage
Chloramphenicol	Hepatotoxicity Bone marrow toxicity Aplastic anemia Carcinogenicity
Ampicillin	Asthma attacks Eosinophilia, leukopenia, and agranulocytosis Cholestatic hepatitis Allergic reactions Exfoliative dermatitis Anemia Thrombocytopenia Thrombocytopenic purpura
Sulfonamides	Skin reactions Blood dyscrasias Carcinogenicity
Penicillin	Anaphylaxis

The accumulation of antibiotics in the human body leads to chronic toxicity. For example, quinolones and tetracyclines used in aquaculture can affect the development of children's teeth (Kümmerer 2009). In addition, residual effects of tetracycline have been found to cause impaired fetal development, gastrointestinal disorders, proinflammatory, cytotoxic, and immunopathological effects on human health (Redwan Haque et al. 2023).

In addition, many epidemiological studies have argued that early life exposure to antibiotics through the food chain or drinking water and vertical maternal transmission are associated with an increased risk of childhood obesity (Cox and Blaser 2015).

#### Allergic reactions

Effects, such as anaphylaxis, skin reactions, and delayed hypersensitivity reactions, can occur when people consume foods of animal origin containing allergenic drug residues. Examples of antibiotics that cause such reactions include residues of other beta-lactam antibiotics, such as penicillin and cephalosporins (Bayou and Haile 2017).

Tetracyclines have caused specific reactions including rash, phototoxic dermatitis and allergy (Redwan Haque et al. 2023). In addition, β-lactams cause allergic reactions in the human body (Davies and Davies 2010). On the other hand, the presence of residues of sulfamethazine, oxytetracycline, and furazolidone is thought to have immunopathological effects in humans (Nisha 2008).

#### Teratogenicity

Any chemical or drug that has a harmful effect on the fetus or embryo during pregnancy results in congenital disorders that affect both functional and structural integrity (Beyene 2016). For example, benzimidazoles and antihelminthics are toxic to the embryo when administered early in pregnancy, and the benzimidazole oxfendazole is mutagenic. Enrofloxacin, a fluoroquinolone antibiotic that inhibits bacteria by targeting DNA gyrase, has been shown to be teratogenic to rabbit and rat embryos (Guzmán et al. 2003).

#### Carcinogenicity

Carcinogenic antibiotic residues are covalently bound to various intracellular components, such as glycogen, glutathione (GSH), DNA, RNA, proteins, and phospholipids, posing a hidden danger (Beyene 2016). Chloramphenicol residues in food have been reported to cause cancer (Shahid et al. 2021).

Additionally, nitrofurans, nitroimidazoles, and quinoxaline interact or covalently bind with various intracellular compounds, such as proteins, ribonucleic acid, glycogen, phospholipids, and glutathione. This effect can lead to changes in cellular components such as DNA (Bayou and Haile 2017).

#### Antibiotic resistance

Sub-therapeutic concentration over a prolonged period of time is a potentially dangerous practice as it is one of the strongest selective pressures leading to the emergence of antibiotic resistance (Myllyniemi et al. 2000).

Antibiotics, particularly those used in the poultry and veterinary industries, increase the chances of bacteria surviving under antibiotic stress by exerting selective pressure resulting in the evolution of multidrug-resistant (MDR) strains. These MDR strains have been widely reported in soil and aquatic environments (Shahid et al. 2021). The main mechanisms of MDR are given in Table 2. The most intuitive negative impact of MDR on human health is that pathogens become resistant to antibiotics used in human medicine, making the disease resistant to standard treatments. For example, Vieira et al. found that resistance in *Escherichia coli* showed a high degree of correlation between food animal (particularly poultry and pig) origin and human origin, suggesting that a large proportion of resistant *E. coli* isolates causing human bloodstream infections may be of food origin (Vieira et al. 2011).

**Table 2:** Main mechanisms of multidrug resistance (Ghimpețeanu et al. 2022)

	MDR mechanism
Cephalosporins, Penicillins, Cefotaxime, Monobactams, Carbapenems	Cleavage by -lactamases, Carbapenems, Cefotaximes, and altered Penicillin-binding proteins
Chloramphenicol	Mutation of the 50S ribosomal subunit, reduced membrane permeability, and elaboration of chloramphenicol acetyltransferase
Ciprofloxacin	Efflux, modification, target mutations
Rifampin	Altered -subunit of RNA polymerase
Erythromycin, azithromycin	Ribosomal methylation
Gentamicin, streptomycin	Ribosomal mutations, enzymatic modification, 16S rRNA methylation, and efflux pumps

Given the health risk issues associated with MDR in food-producing animals, researchers are committed to finding alternatives to the use of antibiotics on livestock farms. Recently, plants and their extracts containing bioactive compounds, organic acids, probiotics, prebiotics, and other natural materials have been studied to replace antibiotics in livestock farms (Suiryanrayna and Ramana 2015; Zhai et al. 2018). For example, phytogenic feed additives, such as black cumin, cinnamon, and their extracts, have been found to be effective in reducing stress, improving the immune system and ultimately increasing the growth rate of animals (Ghasemi et al. 2014; Li et al. 2015; Toghyani et al. 2011). In addition, dried marigold (*Calendula officinalis*) has been investigated as an antibiotic growth promoter in broiler chickens (Foroutankhah et al. 2019). The addition of a processed phytogenic feed additive reduced core body temperature and increased feed intake and water consumption in broiler chickens, resulting in increased body weight compared to control chickens (Greene et al. 2021).

Resistant bacteria can be pathogenic and cause adverse health effects. Resistance genes can be transferred to pathogenic bacteria (Ngangom et al. 2019). The most known antimicrobial resistant strains are antimicrobial-resistant *Salmonella*, macrolide- or fluoroquinolone-resistant *Campylobacter*, glycopeptide- or streptogramin-resistant *Enterococci*, and multiple antimicrobial-resistant *Escherichia coli* (Phillips et al. 2004). Infections caused by *Campylobacter* and *Salmonella* are prime examples of antibiotic use in animals and risk to human health. Humans usually become infected with these microorganisms after consuming food contaminated with these bacteria. DT-104, one of the *Salmonella* strains, and *Campylobacter jejuni* are bacteria that are resistant to many classes of antibiotics. This makes these infections difficult to treat and increases the risk to human health (van den Bogaard and Stobberingh 2000).

#### Microbiota changes

Once in the human body, antibiotic residues can interact with the human microbiome, which includes a large number of different microorganisms living in the human body. 95% of these microbes are beneficial bacteria, while the rest are harmful bacteria and opportunistic pathogens (Ben et al. 2019). In general, the bacteria that live in the gut act as a barrier, preventing incoming pathogens from colonizing and causing disease. Antibiotic residues can reduce the total number of bacteria or selectively kill some important species. Broad-spectrum antibiotics can affect a wide range of gut flora, causing gastrointestinal distress. In addition, antibiotics may reduce the total number of these benign bacteria or selectively kill some important species when consumed in foods containing their residues (Myllyniemi et al. 2000).

Changes in the gut microbiota in children lead to complications, such as obesity and increased risk of allergies, due to (Li et al. 2019). Exposure to antibiotic residues in childhood disrupts the maturation and colonization of the gut microbiota (Chen et al. 2022). Furthermore, disruption of microbiota homeostasis leads to neurological disorders, such as anorexia, depression, Alzheimer's disease, autism, inflammatory bowel disease, and Parkinson's disease (Piñeiro and Cerniglia 2021). Worse still, if gut bacteria develop antibiotic resistance and multiply into superbugs, the diseases they cause will be fatal because they cannot be treated (Ben et al. 2019).

The human gut microbiota is another important factor in the development of antibiotic resistance, especially in hospitalized patients (San Millan 2018). Antibiotic resistance genes present in the gut microbiome can be exchanged between commensal species and horizontally transferred from pathogenic species (Francino 2016). Conjugative plasmids play an important role in the spread of resistance genes in the gut. Resistant clones can be isolated relatively easily from the gut microbiome (San Millan 2018). Antimicrobials should be used with caution to protect or restore the microbiome and/or prevent colonization by multidrug-resistant organisms (Tosh and McDonald 2012). In addition, some studies have shown that antibiotic resistance genes may be present in the human gut microbiome (Sommer et al. 2009).

#### Mixture toxicity

In some cases, more than one antibiotic are used to treat infections or improve growth. Multiple antibiotic exposure is common, especially in aquatic systems. However, in this case, the toxic effects, especially mixture toxicity, caused by multiple antibiotic use should not be ignored (Kovalakova et al. 2020).

Since antibiotics are used together, two or more antibiotics can be used simultaneously. Although the toxic effects of single use of antibiotics are generally elucidated, the situation with mixture toxicity is quite complex (Kovalakova et al. 2020). For example, González-Pleiter et al., in their study evaluating the single and mixture toxicity of five different antibiotics, showed that cyanobacteria, as a target organism, was more sensitive to its toxic effects than green algae, a non-target organism. These results show that the different organisms tested have very different mixture toxicity towards the same mixtures (González-Pleiter et al. 2013). Similarly, Long et al. have shown that antibiotic mixtures can cause different toxic effects on target organisms (Long et al. 2016).

It is obvious that combined antibiotic residues pose a danger, especially for the environment. At this point, the use of sulfonamide group antibiotics together with antibiotics from different groups affects all living things through the food chain, especially aquatic creatures (Białk-Bielińska et al. 2013). Ren et al. also claimed that the residues in water formed by quinolone group antibiotics (ciprofloxacin, norfloxacin, enrofloxacin, lomefloxacin, and their binary combinations) together with different antibiotics may pose a serious problem for the environment (Ren et al. 2021).

Although there are predictions about the toxicity of mixtures of antibiotics, studies on this subject are quite limited. Therefore, further studies are needed to elucidate this issue.

## Reduction of antibiotic residues in foodstuffs

Each country must enact legislation on the use of antibiotics in animals and residue limits in food. The EU banned the use of antibiotics in 2006 to protect animal health (Carlet et al. 2012). In the United States, meat and meat products contain residues of carcinogenic chemicals and/or their genotoxic metabolites. FDA regulations have been effective in preventing allergenic, toxic, and carcinogenic animal drug residues in food. Although the FDA approves some new animal drugs with some requirements: (i) the drug must be used in limited concentrations, (ii) the drug must not be carcinogenic, (iii) no carcinogenic residues can be detected in animal tissues or products after appropriate withdrawal of the drug (Bacanlı and Başaran 2019). FDA prohibits the off-label use of chloramphenicol, furazolidone, nitrofurazone, sulfonamides, and fluoroquinolones in lactating animals. Irrational use of drugs in veterinary medicine should also be avoided (Nisha 2008). Legislation on the control of antibiotic residues in animals and their products is set out in EU Council Directive 96/23/EC. The use of antibiotics below therapeutic doses in food-producing animals is prohibited in EU countries. In Sweden, the use of antibiotics in animal feed was banned in 1985. In Denmark, the use of avoparcin was banned in 1995, followed by virginiamycin in 1998. The EU banned avoparcin in 1997 and four growth promoters, spiramycin, tylosin phosphate virginiamycin, and zinc bacitracin. The Danish food industry stopped the use of all antimicrobials for growth promotion in 1998 (Willis 2000). In 2005, FDA identified fluoroquinolone-resistant *Campylobacter* spp. and banned the use of enrofloxacin in food animals due to increased levels (Huyghebaert et al. 2011). The use of quinolones in animals was banned by the Australian Pesticides and Veterinary Medicines Authority in 2004 (Cheng et al. 2012). However, despite the bans in these countries, most antibiotics are still used in many countries for the treatment and/or prevention of disease and growth promotion in animals (Muaz et al. 2018).

The Maximum Residue Limit (MRL) is the maximum concentration of a chemical that can be present in feed or food (milk, meat, eggs) for human consumption during a given slaughter or harvest, processing, storage and marketing (Lee et al. 2001). Below this limit, scientists and authorities consider that there is no health risk to the consumer and no effect on the production process. The MRL concept strikes a balance between consumer expectations and producer constraints, allowing antibiotics to be used without being banned and in complete safety. The MRL is calculated taking into account both the toxicological risk and the possible impact of residues on the human digestive flora (Boisseau 1993).

The withdrawal period is the time required after administration of a medicinal product to a food-producing animal to ensure that residues of toxicological significance reach a safe concentration in the target edible tissues or milk, as defined by the tolerance. This interval is necessary to minimize or avoid harmful levels of residues in products intended for human consumption (Ngangom et al. 2019).

Only a small portion of foods of animal origin are consumed raw, so cooking is very important to reduce antibiotic residues in foods. However, this process is not sufficient to remove antibiotic residues (Bacanlı and Başaran 2019). In addition, it can be said that different methods can be effective in the reduction of drug residues in food. Fat loss, protein denaturation, altered pH, and water loss have been used to alter the chemical structure, solubility and concentration of drug residues (Javadi 2011). Heating milk by sterilization, pasteurization, or ultra-heat treatment (UHT) has been reported to reduce drug residues in milk. UHT was found to reduce tetracycline levels by 30% and oxytetracycline levels by 40%, while sterilization reduced tetracycline levels by 98% (Zorraquino et al. 2011). Raw eggs are usually used after heat treatment and chilling. Heat treatment promotes protein denaturation, dehydration, and pH changes, contributing to changes in chemical formulation, residue reduction and residue solubility. As a result, the concentration of tetracycline residues decreased by 52% and 47% after boiling and frying eggs, while the concentration of enrofloxacin decreased by 69% and 58%, respectively (Ezenduka et al. 2011). However, when eggs were boiled at 100 °C for 15 min, the concentration of antibiotic residues, i.e. chlortetracycline, ciprofloxacin and enrofloxacin, decreased by 61%, 87%, and 93% respectively, while other residues such as chlortetracycline decreased by 20–22%. Sulfanilamide decreased by 44–49% when eggs were stored in a refrigerator at 10 °C for 4 weeks (Alaboudi et al. 2013). However, in some cases, heat treatment and the use of different cooking methods have shown that such food processing does not completely eliminate residues and that the concentrations of some residual antibiotics may even increase after frying or roasting (Li et al. 2017).

## Determination of antibiotics in foodstuffs

Although it is not possible to accurately detect antibiotic residues in food, it is important to perform analyses to ensure food safety. Before the twenty-first century, methods, such as microbial growth inhibition assays, microbial receptor assays, enzymatic colorimetric assays, receptor binding assays, chromatographic methods, and immunological tests, were commonly used to test for antibiotic residues in foods (Mitchell et al. 1998). However, as all these methods are based on the analysis of a small number of samples, new methods are not available. The need for development is clear. An ideal method for detecting antibiotic residues in food should be rapid, effective, and inexpensive, and should meet the qualitative and quantitative requirements for a wide range of antibiotics (Chen et al. 2019). To ensure food safety for consumers, more and more work is being done to find effective and rapid methods for detecting antibiotic residues in food.

Basically, qualitative, quantitative, and semi-quantitative analytical methods are used to determine the presence of antibiotic residues in food (Mitchell et al. 1998). At this point, sample preparation procedures are the cornerstone of the instrumental analytical process due to the complex matrices in food samples (matrices with high fat, oil, starch, protein or sugar content) and concentrations at the level of nanograms per liter or nanograms per gram or even lower (Freitas et al. 2014; Frenich et al. 2014). In addition, the analytical methods used to determine antibiotic residues in food can generally be divided into screening analytical methods and confirmatory analytical methods. Screening methods use readily available equipment to determine the amount of one or more antibiotics, whether or not the antibiotic is measured. Methods in this category include microbial inhibition, enzyme immunoassay, rod format (lateral flow), radioimmunoassay (RIA), chemiluminescence immunoassay (CLIA), fluorescence immunoassay (FIA), and colloidal gold immunoassay (CGIA). For confirmatory methods, analytical techniques have gradually improved. These methods require the use of expensive equipment (LC–MS/MS, GC–MS/MS) and highly skilled personnel (Ghimpețeanu et al. 2022). Of these methods, LC–MS/MS in particular can confirm more than 100 target antibiotics of different classes in a single run if the pre-treatment method can remove as many antibiotics as possible from the food samples and measure antibiotic residues at low nanograms per liter or nanograms per nanogram (Chen et al. 2019).

Fully automated biosensors are becoming increasingly important in the detection of antibiotics in food. This method is fast and specific due to the biorecognition element used. However, it has some limitations, such as the instability of the biosensor component and the size of the physicochemical transducers used in biosensors (Bacanlı and Başaran 2019). Research into biosensors for the detection of antibiotic residues has been increasing since the 1980s. The fact that biosensors are selective, inexpensive and provide rapid results has attracted the attention of the food industry. Antibiotic residues in various foods, such as milk, eggs, and meat, can be reliably determined using the biosensors developed (Gaudin 2017).

## Conclusion

Unlike other routes of exposure, consumers cannot know whether the food they eat contains antibiotic residues. This situation highlights the need for intensive studies on antibiotic residues in food. At this point, both consumers and producers need to be aware, and the authorities need to be sensitive to the regulations and take strict measures to ban the use of antibiotics and review MRLs when necessary. In particular, as recommended by the World Health Organization (WHO), it is very important to avoid unnecessary use of antibiotics and to consider the risk–benefit ratio when using antibiotics in order to prevent toxic effects caused by antibiotic residues and the development of antibiotic resistance. In addition, further research into the use of alternatives, such as herbal substances and probiotics, could be considered to limit the use of antibiotics.

## Data Availability

There is no.
